# Parents’ experiences of a health dialogue in the child health services: a qualitative study

**DOI:** 10.1186/s12913-019-4550-y

**Published:** 2019-10-30

**Authors:** Linda Håkansson, Mariette Derwig, Ewy Olander

**Affiliations:** 1 Center of Excellence for Child Health Services, Baltzarsgatan, 31 205 02 Malmö, Sweden; 2 Department of Health Sciences, Faculty of Medicine, Lund, University, Lund, Sweden; 3 Blekinge Institute of Technology, Department of Health, Karlskrona, Sweden

**Keywords:** Child health care nurses, Child health services, Health dialogue, Health promotion, Participation, Parents’ experiences, Qualitative interviews

## Abstract

**Background:**

The Child Health Services in Sweden is a well-attended health promoting setting, and thereby has an important role in promoting healthy living habits in families with young children. Due to lack of national recommendations for health dialogues, a Child Centred Health Dialogue (CCHD) model was developed and tested in two Swedish municipalities. The aim of this study was to explore parents’ experiences of health dialogues based on the CCHD model focusing on food and eating habits during the scheduled child health visit at four years of age.

**Methods:**

A qualitative design with purposeful sampling was used. Twelve individual interviews with parents were conducted and analysed with qualitative content analysis.

**Results:**

The analysis resulted in three categories: The health dialogue provides guidance and understanding; Illustrations promote the health dialogue; and Space for children and parents in the health dialogue. In addition, analysis of the latent content resulted in a single theme reflecting the parents’ voice on the importance of having a health dialogue on food and eating habits. The health dialogue, promoted by illustrations, provided guidance and understanding, and gave space for children’s and parents’ involvement.

**Conclusions:**

The results indicate that health dialogues using the CCHD- model create supportive conditions for family members’ active participation in the visits, which may strengthen empowerment and health literacy. The study provides knowledge and guidance for further development, evaluation and implementation of the model.

## Background

The Child Health Services (CHS) are ubiquitous throughout Sweden and reach almost all children aged 0–5 years and their families, and the CHS thereby provide an important health promotion setting at the individual as well as the population level. The CHS aim to promote good health on equal terms for all children through universal and indicated activities, including health dialogues, health examinations, immunisations and parental support. This is outlined in the National Child Health Programme, which is based on guidelines from the National Board of Health and Welfare [1]. One of the obligations of the CHS is to promote a healthy lifestyle to improve children’s physical and mental health and wellbeing, and prevent childhood obesity and associated diseases later in life through recurrent health dialogues and early identification of risk factors [2]. Childhood obesity is five times more common today than two decades ago [3]. Obesity acquired during preschool years is likely to be persistent, and obese children are likely to become obese adults [4, 5]. Thus, CHS professionals have an important mission to offer parents and children guidance about healthy living habits early in the child’s life [6].

Previous studies indicate that health dialogues with parents can have a positive influence on children’s living habits [7]. Parents express that they want their children to have a healthy lifestyle, and therefore consider it important to have a dialogue about healthy living habits during their CHS visits. Healthy eating habits is a subject the parents would particularly like to discuss. Through such dialogues, parents often become aware of their child’s eating habits and are open minded about promoting them, but perceive it difficult to manage them in daily life [8].

At present, there are no national guidelines for the execution of health dialogues within the CHS, and practices can differ [7]. There is therefore a need for uniform models for CHS health dialogues that can strengthen family participation, health knowledge and empowerment in accordance with the aim of an equal and equitable CHS. This was the impetus for creating the Child Centred Health Dialogue (CCHD) model [9], which was developed by two of the authors (LH and MD) based on a health promoting and supportive environment approach [10] (Table 1). The development was guided by the Medical Research Council’s framework for complex interventions consisting of four key elements: development, feasibility, evaluation and implementation [11].

**Table 1:** The Child Centred Health Dialogue model (created by authors LH and MD)

The Child Centred Health Dialogue model			
The CCHD- model consists of two parts, a universal part directed to all 4-year-olds and their families visiting the CHS and a targeted part to families where the 4-year-old is identified with overweight or obesity. The universal part consists of a structured health dialogue between the nurse, the parents and the 4-year-old using eight illustrations. The first four illustrations focus on fruit and vegetable consumption, intake of sweetened beverages and total energy intake. The remaining illustrations focus on other important behaviors associated with children’s health and body weight, such as physical activity, tooth brushing and sleep. The CHS nurse introduces each illustration to the child first, followed by an open dialogue with the child and the parents, their reflections and questions. After each illustration they summarize the conversation together and recap the health issues important for the individual family (Rikshandboken för barnhälsovård, 2019) The purpose of the CCHD- model is to strengthen empowerment, promote participation and health literacy with a structure based on the following items: approach, dialogue, tools and process.			
Approach	Dialogue	Tools	Process
Salutogenesis Empowerment Alliance/Partnership Relationship Neutrality Non-judgemental Trust	The child and the parents’ questions Open questions Contextually Dialogue and participation Reframing	Neutral educational illustrations Iso-BMI chart	Identifying protective factors and family resources Promoting and strengthening HL Small changes in lifestyle with solution-focused approach Long-term goals

The CCHD model rests on four theoretical foundations: person-centred approach, participation, health literacy and empowerment. Using a person-centred approach, the family’s story is a starting point for the health dialogue and a first step in a partnership between the nurse, child and parent [12, 13]. To grasp the child’s perspective, in accordance with the United Nations Convention on the Rights of the Child [13], the child must be given the opportunity to express her or his thoughts, feelings and experiences [14]. Participation is thereby a fundamental component of health promotion in the CHS, characterized by involvement of children as active partners, which enhances the families’ understanding of their own needs and desires [15, 16]).

Strengthening health literacy in a health promoting setting such as the CHS comprises both enhancement of individual health literacy and creation of health literacy supportive environments [17]. Health literacy differs among families, hence there is a varying capability to understand and use health information, to communicate and to make judgements and decisions in everyday life in order to maintain, promote or improve health [17, 18]. Customized health communication in plain language with brief counselling approaches can improve parents’ health literacy [17, 19]. According to Trollvik [20] children’s health literacy can be improved with a complementary use of educational illustrations, since children often become involved in stories and pictures.

From a family perspective, empowerment can be described as a process where people’s opportunities are supported and promoted in order to help individuals meet their own needs, solve their own problems, and mobilize their resources [21]. To strengthen empowerment, a health dialogue should focus on the resources and knowledge the families already possess, rather than on what they lack or need.

Moreover, a health dialogue with a person-centredapproach, participation and strengthening of health literacy, improves the conditions for development of empowerment [12, 13, 15, 17, 21] and may therefore be an essential cornerstone in health dialogues with children and parents in the CHS.

As a first step in the implementation process [11], the CCHD model was introduced and tested at three pilot CHS units in two municipalities in Sweden. Six nurses at these centres received theoretical and practical training about the CCHD model as well as recurring mentoring dialogues during the implementation of the model. In this feasibility part of the implementation process, an essential task was to catch the experiences of all participants in the health dialogue – nurses, parents and children – in order to explore various perspectives of the model before expanding to implementation. A previous study of CHS nurses’ experiences of working with the CCHD model showed that nurses felt more comfortable in the dialogue about healthy food habits and found the children to be more talkative and involved [22]. The aim of this study was to explore parents’ experiences of health dialogues in the CHS based on the CCHD model focusing on food and eating habits during the health visit when the child is four years old.

## Methods

In this study a qualitative inductive approach with individual interviews and qualitative content analysis was used to examine parents’ experiences of the CCHD model in the CHS [23, 24].

### Setting and participants

The study was conducted at three pilot CHS units in the two municipalities in southern Sweden where the CCHD model had been introduced and applied. Four CHS nurses, two in each municipality, had applied the CCHD model for at least six months.

The municipalities consisted of a mixed rural and urban population of approximately 12,000 and 10,000 residents, respectively and were similar with respect to social factors such as level of education, mean age and proportion receiving social welfare benefits [25].

Parents who had attended a 4-year health visit and participated in health dialogues based on the CCHD model during September to December 2015 were eligible. A stratified purposeful sampling method was used to optimize variation in the study groups with respect to CHS nurse and time of exposure to the CCHD model [23]. This selection procedure contributed to a consistent distribution between the nurses and throughout the time period, and possibilities to share the parents’ experiences regardless of which nurse they had met. In total, twelve parents of twelve children were contacted by telephone, six from each municipality, three from each CHS nurse. All parents initially contacted, agreed to participate in the study. In accordance with ethical guidelines for research and the ethical approval for the study, the parents received information about the study, the interview procedure and confidentiality of their data by e-mail and orally at the beginning of each interview as a basis for providing their informed consent.

### Data collection

Data were collected by the first author (LH) over a two month period, three to seven months after the health visit. A semi-structured interview guide was developed by authors LH and EO. The guide included nine questions with suggestions for further cues to encourage more in-depth responses when necessary [23] (see Additional file 1). The question areas included how the participants experienced the health dialogue and illustrations, how relevant and useful they thought these were, and how the parents experienced their own and their children’s opportunities to participate. Before use in the interviews, the guide was tested by interviewing two child health nurses active in research, after which minor changes were made. All twelve interviews were conducted by LH. The first two interviews were used as a pilot of the interview guide and procedure. These were analysed with regard to the depth and relevance of the responses in relation to the aims of the study, and were found to be adequate and were therefore included in the analysis together with the following interviews. After analysis of the tenth interview, nothing new was found to emerge. For confirmation, another two interviews were performed and indicated saturation of the data. The parents were informed that the interviewer was a Child Healthcare Coordinator and district nurse with work experience from the CHS, but were uninformed that she was involved in the development of the CCHD model. LH had pre-understanding as she participated in the development of the CCHD model and had experience of meeting children and parents as a CHS nurse in other municipalities. There was no relationship between the parents and the interviewer before study commencement.

Nine interviews were conducted in the family’s home and three at the municipal library. The children were present at ten of the interviews. The interviews lasted between 25 and 35 min. All interviews were recorded on an iPhone and transcribed verbatim. After transcription, the data were given code numbers in order to assure the informants’ confidentiality.

### Data analysis

A qualitative content analysis based on Graneheim & Lundman’s [24] model with manifest and latent analysis was used. The manifest analysis followed several steps in accordance with Lundman and Hällgren-Graneheim (Table 2) [26]. The first author transcribed the interviews and read the material several times in order to create a sense of entirety. Meaning units were then identified, condensed and coded based on their conformity and differences. The codes were brought together into sub-categories and lastly into categories, in order to reach a higher level of abstraction reflecting key messages as well as expressing the manifest content of the text. Thereafter, in the latent analysis, the underlying meaning of the content in the categories was formulated into a theme. To ensure credibility, all steps in the analysis procedure were carried out in a dialogue between authors LH and EO.

**Table 2:** Example of the analysis process; meaning unit, condensations, codes, subcategories and categories (Graneheim & Lundman, 2004)

Meaning unit	So great that the nurse talked mainly with my daughter and it turned out during the conversation that my daughter talked about how things actually are in our home
Condensation	So good that the nurse talked mainly with my child who during the conversation told her how things are at home
Code	Talked mainly to my child
Sub-category	My child in the centre
Category	Space for children and parents in the health dialogue

## Results

Of the participating parents, eleven were female and one was male. The average age was 35 years. For two of the parents, this was their first child, while the remaining ten parents had between one and six previous children. The parents expressed that the health dialogue based on the CCHD model, focusing on food and eating habits during the 4-year health visit, was important and rewarding. The manifest analysis resulted in ten subcategories divided among three categories and the latent analysis identified a theme from an underlying implication of the importance of a dialogue about food and eating habits, from both an individual health perspective and a societal perspective (Table 3).

**Table 3:** Sub-categories, categories and theme

Sub-categories	Categories	Theme
Talking about food and eating habits is important and relevant Uncomplicated conversation gives guidance and understanding Reflecting on eating and drinking in everyday life Support and confirmation Wishes for the health dialogue	The health dialogue provides guidance and understanding	It is important to have a dialogue about food and eating habits, − it’s about our children’s health
Illustrations make it easy to understand Illustrations open up	Illustrations promote the health dialogue	
My child in the centre Important to involve the child in the right way Parents’ opportunity to participate	Space for children and parents in the health dialogue	

### The health dialogue provides guidance and understanding

#### Talking about food and eating habits is important and relevant

The analysis showed that although parents felt aware of the importance of healthy food and eating habits, talking about this at the 4-year health visit at CHS was considered important and gave the opportunity to discuss perceived problems. Even when the issue discussed during the health dialogue was not experienced as relevant for their own family, it was considered relevant and important for other families lacking this knowledge.

“It’s great that the CHS has a dialogue about food because it can be quite difficult, especially when the children grow older and get fussy with food.” [Parent 4]

#### Uncomplicated conversation gives guidance and understanding

The parents found the dialogue about food and eating habits easy to understand and enjoyable. They received support and encouragement, and both children and parents learned new things. Most parents were worried that their child did not eat enough. In the dialogue they got a better understanding of what could be enough food for their child.

“I asked how much children need to eat, the nurse told me that a benchmark can be the child’s palm, a hand of each (potatoes, vegetables and meat). I realized that my child does that and it became clear. [Parent 3]

#### Reflecting on eating and drinking in everyday life

The health dialogue was also experienced as reflective with discussion without judgement on eating and drinking in everyday life. The parents appreciated the nurses’ normalization of children’s ways of avoiding certain food and various other eating habits. According to the parents their worries were diminished and they listened more to their child and argued less about food.

“We talked about feeling full enough. Now I know that if my child says that he is full, it’s enough.” [Parent 2]

#### Support and confirmation

The CHS was experienced as a permissive setting for bringing up daily life struggles with food and eating habits and for being taken seriously. It felt important to come to the health visit and talk about children’s health. The dialogue gave support and confirmed the parents in promoting good food and eating habits and limiting sweets and sweet beverages.

“It is hard to resist relatives who provide comments on why he doesn’t get sweets so often. Therefore the dialogue with the nurse felt very good.” [Parent 5]

#### Wishes for the health dialogue

Along with the positive experiences some parents also expressed a few wishes for the health dialogues. Some parents experienced the dialogues as a little too shallow. They desired a more exploring approach with open questions to stimulate the child’s participation in the dialogue even more. Some parents felt that a part of the dialogue should be addressed more to themselves. As they are responsible for their children’s health, they need a better understanding. A desire for the possibility to come separately for the health dialogue was also expressed by parents who didn’t live together.

“I would have liked a part of the dialogue to be addressed to me as a parent, because we must have an understanding.” [Parent 6]

Furthermore parents asked for additional health information such as illustrations to bring home and continue the dialogue in the same way as they did at the health visit.

### Illustrations promote health dialogue

#### Illustrations make it easy to understand

The analysis showed that parents appreciated the use of pedagogical illustrations in the health dialogue and believed that these made it easier for the children to talk, understand and follow the dialogue. The illustrations were experienced as uncomplicated, and could not be misunderstood.

“It was fun for both me and my child. The illustrations used in the dialogue could not be misinterpreted and it was easier to ask questions. It felt like a real hit.” [Parent 3]

#### Illustrations open up

The parents experienced that the illustrations invited the child to take part and opened up the dialogue. Usually the nurse talked mostly with the parents about food and eating habits. According to the parents, the illustrations changed the health visit with a better opportunity for the children to participate. They became very talkative and voiced their opinions, thoughts and questions. The illustrations were exciting and even children who used to be shy would talk and show that they knew and understood more than the parents believed.

“My child is very wise and verbal and went all in and analysed all the illustrations, ha, ha. She participated in the dialogue and had many thoughts.” [Parent 10]

### Space for children and parents in the health dialogue

#### My child in the Centre

It became obvious that the parents often chose to take a step back and let the child be in the centre in the health dialogue. It was important that the focus was on the child but also that the parents had the opportunity to add, confirm or clarify things, and support the child if needed.

“I was silent and let them talk. He felt so mature and grew so much in front of me when they talked together, he understood everything. I got a new perspective, great fun.” [Parent 1]

#### Important to involve the child in the right way

The parents thought it was important to involve the child in the dialogue about food and eating habits and enjoyed the way the nurse did it. The parents expressed that the nurse seemed to listen carefully to the child, gave affirmative answers and talked with the child in an understandable way. Instead of talking about unhealthy food and eating habits, she gave suggestions about what was healthy to eat regularly and what to eat and drink more rarely.

“It is important that children participate in the dialogue but it is delicate how we talk about food with them.” [Parent 8]

#### Parents’ opportunity to participate

The analysis showed that the parents described the nurses as skilled in involving parents in the health dialogue. They showed interest and listened to questions and concerns of the parents, and gave a lot of space for reflections and discussion. Parents described this as important and they felt participatory and never found it difficult to talk with the nurse.

“I felt I got a lot of space and felt that we were talking together. Sometimes I talk so much and will then get a reminder that the nurse may also want to say something ha ha ha.” [Parent 11]

### Theme

The theme “*It is important to have a dialogue about food and eating habits, it’s about our children’s health”* reflected the underlying content that recurred in the analysis. Parents valued the health dialogue highly both for their own family and for other families. The parents emphasized the importance of talking about food and eating habits at the CHS from different aspects based on their own family’s concerns, questions and worries about food and eating habits and their child’s health. They expressed that the health dialogue was important with respect to current trends in society with unhealthy food and eating habits.

“From a social and health perspective, I consider it is crucial and increasingly important for the CHS to talk about food and eating habits. There is a great need for knowledge among families.” [Parent 2]

## Discussion

The overarching theme of the study illustrates the parents’ positive opinions on the value of the health dialogues based on the CCHD model, both from an individual health perspective and from a public health perspective. According to the parents’ experiences, the CHS is an important setting for health promotion and disease prevention. It creates a supportive environment for continuous health dialogues on lifestyle issues in accordance with the Child Health Care aims (National Board for Health) [1], the Swedish national public health goal [27] and Health 2020 [28] emphasizing healthy food and nutrition throughout the lifespan.

This study, like previous studies [8, 29], shows that parents want to promote healthy habits but need professional guidance and hands-on advice for managing difficulties experienced with food and eating habits in everyday life. The results indicate that the health dialogue based on the CCHD model during the 4-year health visit provides such guidance and understanding. Some parents experienced the health dialogue as a little too shallow and some wanted a part of the dialogue more clearly addressed to them. To be able to understand recommendations given during a health dialogue, parents sometimes ask for deeper dialogue related to health and ill health [8, 29]. These findings illustrate the importance of being attentive to what individual parents want to talk about, be contextual and provide support and guidance for the family.

A common concern among the parents was that their children ate portions that were too small. Studies by Håkansson [22] and Regber et al. [30] also showed that parents felt worried as well as anxious that their children might develop underweight. The parents in our study expressed that they became less worried after the health dialogue and more responsive to their children’s appetite, and let the children decide more about how much they wanted to eat. Such actions are important for the development of the child’s self-regulation and promote a healthy lifestyle [31]. This indicates that the parents felt trust in the nurse and in the suggestions discussed in the dialogue.

The illustrations used in the health dialogue were found to be easy to understand and invited the children to become active in the dialogue. By using illustrations, children can be involved at a level congruent with their cognitive and social skills and given space in the dialogue [20]. Children have their own way of understanding and constructing the environment based on their own experiences [32]. In a dialogue supported by illustrations, the adult encodes the health message around a story, which increases the child’s ability to understand and may thereby promote health literacy [33]. In this way the illustrations contribute to what Hydén and Baggens [34] describe as “a joint working relationship” between the parties.

The parents’ desire for additional health information for a continued health dialogue at home indicates the need to develop information that is easy to understand and applicable to the family’s own food and eating habits. At the time of the study there was no additional health information such as a booklet or an application for mobile phone or tablet related to the educational illustrations available in the CCHD model. In order to strengthen the possibility of continued health dialogue at home, a storybook was created after this study.

The results show that the nurses created space for both children and parents in the health dialogue and that the dialogue was child centred [35]. According to Sommer [31] and Coyne et al. [36] such an approach is needed in health care, as children’s participation is necessary for the possibility to share their experiences, perceptions and understanding. Even the nurses in the CHS experienced that health dialogues based on the CCHD model became easier and better when the children and parents were more active involved in the dialogue [22].

The results also show that the parents experienced that the conversation was understandable and useful for the family. They felt it was based on their own and their child’s descriptions, questions and thoughts, the family’s situation, focusing on their strengths and abilities. Such health dialogues promote empowerment [35] but also strengthen a functional as well as a comprehensive health literacy [19] with the capacity to understand and use health information and to make informed and reasoned choices, as outlined by the WHO [18]. In this way, health literacy does not automatically lead to empowerment [37]. Health dialogues, as in the CCHD model, have to embrace space for questions and reflections, to enhance critical consciousness, self-esteem, and self-efficacy [37, 38].

The study indicates that the CCHD model enables children’s participation with a child perspective in line with the Convention on the Rights of the Child [14]. In this way a health dialogue based on the CCDH-model is consistent and corresponds to a person- and child centred health care [12], and the new Patient Act, enacted in Sweden in 2015 (2014:821), emphasizing that the child should be an active party in decision making, and should receive relevant and age-appropriate information [39].

The interviews were conducted three to seven months after the CHS visits; this was a conscious choice as it takes time to introduce new habits and for changes to take place. This postponement of the interviews may have contributed to some recall bias, but none of the parents mentioned any difficulties in remembering details of the CHS visit. Very few negative comments were provided by the parents. This could be due to a social desirability bias, as the participants had knowledge that the interviewer was employed within the CHS. However, the interviewer’s role as developer of the CCHD model was unknown to the parents.

Karlsson [40] states that there are pros and cons of being an internal investigator. The first author was well acquainted with the CHS’ activities and environment, and with the CCHD model regarding purpose, implementation and how the dialogue is supposed to be accomplished, which may have added to the credibility of the results. A potential risk was that LH, through her pre-understanding, could have toned down failures and overemphasized what has been good [40]. To minimize this risk, LH’s awareness of her pre-understanding was repeatedly reflected on and discussed with EO to achieve transparency in the material [23]. This reflexivity, together with the triangulation achieved through review of all findings by two authors with different professional backgrounds, may have further contributed to the study’s credibility.

To ensure the trustworthiness of the study, the analysis was performed with frequent back-reference to the aim, the interview guide and consistency to the method described by Graneheim and Lundman [24]. Quotations that illustrate the parents’ statements were included in the results, which may assist the reader to assess the trustworthiness and credibility of the study. According to Polit and Beck [23], it is the reader who can determine the transferability and to what extent and in which context the results can be applied.

This study was developed as a first examination of parents’ experiences of exposure to the CCHD model. The recruitment procedure was conceived to gather a group of parents that was reasonably representative of the families attending the CHS in the respective municipalities, during the time period for the study. As parent ethnicity, gender and lifestyle factors were not taken into account, this may have affected the representativeness of the results. However, the aim of the present study was to investigate the parents’ experiences of the CCHD model and not to compare experiences between different groups. This would be a natural continuation for future studies of the CCHD model.

The presence of children during the interviews gave some interruptions that could have jeopardized the contact between the participant and the interviewer. However, the interviewer never felt that the presence of the children disturbed the interviews.

## Conclusions

The study indicates that health dialogues using the CCHD model at the 4-year health visits at CHS are highly valued by the parents, both from an individual family perspective and a public health perspective. The health dialogue with illustrations creates supportive conditions for parents’ as well as children’s active participation in the health visits and understanding of the health dialogue content, thereby strengthening health literacy and empowerment. In this way the CCHD model adheres to several Swedish and international documents and strategies on health promotion and disease prevention as well as to the Convention on the Rights of the Child [14], and might be useful not only at Swedish the CHS but also in other child health promotion contexts.

Further research and development of the CCHD model should include investigation of determinants such as ethnicity, health literacy and living habits among the participating families, as well as the children’s experiences and perceptions of the CCHD model. This could provide a more thorough understanding of the execution, value of such health dialogues with regard to the children’s living and eating habits, information that is necessary before decision makers can consider implementation of the CCHD model in the Swedish Child Health Program.

## Supplementary information

**Additional file 1.** CCHD Interview Guide.

## Data Availability

The data set generated and analysed during the current study will not be shared to maintain participants’ anonymity and confidentiality but are available from the corresponding author on reasonable request.

## Abbreviations

CCHD: Child Centred Health Dialogue
CHS: Child Health Services
