# Supplementary material for: Parents’ experiences of a health dialogue in the child health services: a qualitative study
Source: BMC Health Serv Res. 2019 Oct 30;19:774. doi: 10.1186/s12913-019-4550-y (PMC6820984; doi:10.1186/s12913-019-4550-y)
Supplement: Supplementary file 1 — Additional file 1. CCHD Interview Guide. [file 12913_2019_4550_MOESM1_ESM.docx]

**Initial dialogue/questions:**

Thank you for taking the time to participate in this interview. You have been asked to contribute because you have recently been to a 4-year child health visit at your CHS. Nine questions will be asked and as you answer the questions you may be asked to develop the answer or describe more. It is completely voluntary to participate in the interview and you can stop the interview at any time if you do not want to participate. The interview is recorded on an iPhone and saved on a USB-drive which is stored in a locked cabinet. All information is treated confidentially and it will not be possible for anyone to identify you. Have you been to a health visit at this CHS before the 4-year visit? Would you like to tell us about your 4-year visit and how long ago it was you were there? At the health visit, the nurse had a health dialogue about eating habits and showed different pictures that you talked about.

- Tell us how you experienced the dialogue about eating habits at the 4-year visit to the CHS.

*can you develop, would you like to describe?*

- Describe what you thought about the content of the health dialogue regarding the conversation and the illustrations about food.

*can you develop?*

- How relevant do you think the health dialogue about eating habits was?

*can you develop, would you like to describe?*

- Tell us how you experienced your opportunity to participate and make your voice heard in the conversation about eating habits during the visit.

*can you develop, would you like to describe?*

- How did you experience your child's involvement in the dialogue when the illustrations were used?

*can you develop, would you like to describe?*

- What did you find was easy in the dialogue?

*can you develop, would you like to describe?*

- What did you find difficult in the conversation?

*can you develop, would you like to describe?*

- In what way do you think what you talked about is useful for you and your family?

*can you develop, would you like to describe?*

- Tell us what thoughts you have about how the conversation will affect your family’s eating habits in the future?

*can you develop, would you like to describe?*

Closure

Do you have any other thoughts?

Is there something you would like to add?
